# The paradox of longer sperm telomeres in older men’s testes: a birth-cohort effect caused by transgenerational telomere erosion in the female germline

**DOI:** 10.1186/s13039-016-0224-1

**Published:** 2016-02-08

**Authors:** Reinhard Stindl

**Affiliations:** apo-med-center, Alpharm, Plättenstrasse 7-9, 2380 Perchtoldsdorf, Austria, Europe

**Keywords:** Intergenerational telomere erosion, Birth-cohort effect, Telomere lengthening, Telomerase, Alternative lengthening of telomeres, Telomeres

## Abstract

Longer telomeres in the somatic cells of an individual have been regarded as a marker of youth and biological fitness within a population. Yet, several research groups have reported the surprising findings of longer telomeres in the germ cells of older men, which translated into longer leukocyte telomere length in their offspring. Although all these studies were purely cross-sectional, a longitudinal trend in the aging testes of individual males was taken for granted. Recently, a high-profile study reported a negative birth-cohort effect on leukocyte mean telomere length in human populations, namely the progressive loss of telomeric sequence between healthy human generations. This is what I based my theory of telomere-driven macroevolution on, 12 years ago. On the basis of published data on telomere length inheritance, I identified the source of human intergenerational telomere erosion in the female germline. Accordingly, because of the resulting birth-cohort effect, there is no need for any paradoxical telomere lengthening in older men’s gonads to interpret the old-father-long-telomered-offspring data.

## Background

Telomeres are the protective caps of eukaryotic chromosome ends and are known to shorten in the dividing cells of somatic tissues of higher animals during aging, in contrast to telomeres in the germline, which have been thought to remain stable in a species. Longer telomeres in the cells of somatic tissues of an individual, compared to other individuals of the same species, have been regarded as a marker of youth and biological fitness. Hence, it came as a surprise, when several labs found longer telomeres in the spermatozoa of older men, which translated into longer leukocyte telomere length in their offspring [1–3]. It was quickly postulated that the paradoxical phenomenon must be caused by biological mechanisms that operate in the testes during a male’s lifetime. Some speculated on telomere elongation in the aging testes [2], others preferred the idea of selection for long-telomered spermatozoa during aging [3]. For whatever reasons, the strange theory of a rejuvenation of the gonads of elderly men has become the exclusive way of thinking among colleagues in the field [4], although the remote possibility of a birth-cohort effect had already been briefly mentioned in a pioneering paper in 2005 [1].

Just recently, I became aware of another new cross-sectional study by Antunes et al. [5], which again claims that telomere length increases in spermatozoa during male aging, without any longitudinal data. The authors concluded further that the alternative telomere lengthening mechanism (=ALT), instead of the enzyme telomerase, must be involved, because of the significant gain in mean telomere length and, even more importantly, because of the increased variation in telomere length seen in the spermatozoa of older male individuals [5]. Antunes et al., like many other colleagues, seem not to be aware of an alternative explanation [6–8] or of recent study findings confirming a birth-cohort effect [9], which obviously represents the source of long-telomered sperms in elderly males [6, 7, 10]; thus, there is no need for any paradoxical telomere lengthening in older men’s testes to interpret the data. Similarly, the reported increase in the variability of telomere length in the spermatozoa of older men [5] can be easily explained by the lifelong mitotic activity of several populations of germ stem cells, which are exposed to environmental insults and subject to all kinds of errors, analogous to the increasing burden of acquired mutations in the spermatocytes of aging men.

## History and current state of the hypothesis

Almost 12 years ago, an evolutionary theory based on intergenerational telomere erosion was introduced [8] and was widely covered by the press [11, 12]. In 2011, based on published data on human telomere length inheritance, I refined my theoretical framework and located the source of human intergenerational telomere erosion in the female germline [6, 7, 10]. According to this model, telomeres in the testes of elderly males are longer than those in young males because the seniors are members of a previous generation (=birth-cohort effect) and therefore skipped, on average, one female-based intergenerational telomere loss. In 2014, I further developed the model of telomere-driven macroevolution and presented a complete biological framework for the old European model of saltatory evolution of nonadaptive characters [6].

In agreement with this theoretical model, a high-profile study, published in the August issue of *Aging Cell* in 2015, finally confirmed the long-awaited birth-cohort effect on leukocyte mean telomere length in humans [9, 10]. The authors found clear evidence that newborns of previous generations must have had longer telomeres, but could not explain the phenomenon. They just speculated that intergenerational telomere erosion could be the consequence of some kind of environmental factor, but it must be a recent phenomenon. Interestingly enough, Holohan et al. found evidence of intergenerational telomere erosion in all the human birth-cohorts they looked at, back to 1920 [9]. Thus, I think it is time that we face the unthinkable, namely that biological evolution is not only about survival of the fittest, but every species becomes unfit after some time, due to an intrinsic cause [6–8].

As already introduced in 2011 and 2014, I propose that the paradoxical findings of longer telomeres in the testes of very old men are the result of transgenerational telomere erosion in the female germline, causing a significant birth-cohort effect in sperm telomere length [6, 7]. Replicative telomere erosion is thought to take place during embryogenesis, when the female primordial germ cells multiply, according to the production-line hypothesis [13]. Starting at puberty, there is a hierarchy with long-telomered oocytes ovulating first [14, 15]. This contrasts with the constantly high telomerase levels in the testes, which ensure lifelong telomere length stability in male germ cells per generation [6, 7]. For a detailed explanation and figure of the proposed patterns of telomere dynamics in the germline of both sexes I refer readers to my previous publications [6, 7, 10]. In the following, I just want to highlight two important observations/study results, on which I initially based my theoretical model and explain them in more detail.

The majority of oocytes of women aged 40 years and older may be aneuploid [16]; this is by far the highest aneuploidy rate of all human cell types during aging [17], with the exception of cancerous cells*.* Critically short telomeres are known to result in telomere associations/fusions and subsequently in numerical and/or structural chromosomal aberrations in dividing eukaryotic cells (=aneuploidy). Thus, the widely known strong positive correlation between the mother’s age at conception and trisomic pregnancies (e.g. Down’s syndrome) [18] seems to be a clear indication of telomere erosion in the female germline [7], and even more so since the father being of an advanced age does not increase the incidence of chromosomally abnormal offspring, despite lifelong germ cell divisions [18]. Although, David L. Keefe had already introduced the telomere theory of reproductive senescence in women in 2006, he has since insisted on an embryonic telomere reset between generations, neglecting any possible intergenerational telomere loss [19].

Further critical evidence, on which I based my model, came from a large human study spanning three healthy generations, published in *PNAS* [20]. Eisenberg and colleagues found that the positive telomere effect of older fathers (age at conception) is cumulative between generations. In the paternal line, older grandfathers (advanced age when the father was born) contributed as much to longer leukocyte telomeres in grandchildren as older fathers did. However, it was found that a grandfather’s positive effect on grandchildren’s telomere length diminished in the maternal line, which clearly supports my concept of female-based transgenerational telomere erosion in the human germline [6, 7]. Accordingly, in this multigenerational study grandfathers’ chromosomes underwent female-based telomere erosion in the maternal line only, because in the paternal line they do not go through female germ cell divisions. Therefore, the positive age effect on telomere length could only be stably transmitted to the grandchildren of the paternal line [6].

## Response to recent criticism and seemingly conflicting data

One of the arguments in the recent *Aging Cell* study regarding the proposed recentness of the phenomenon was that the amount of intergenerational telomere loss observed is too high to be compatible with the survival of the human species for thousands of years [9]. Yet, in accordance with my evolutionary theory, I see the extreme loss as a result of the advanced (and still increasing) age of mothers at the birth of their first child in modern and developed societies, whereas, in ancient human societies, as is the case for all higher animals, girls (females) usually become pregnant starting at puberty. Accordingly, I conclude that the amount of intergenerational telomere loss in all human societies was lower a few hundred years ago and is lower in current relatively undeveloped human societies, analogous to higher animals. This, however, implies that the proposed progressive intergenerational telomere loss will be harder to detect in animal experiments. Yet, I am convinced that the human female age effect is a proof of principle, a ubiquitous principle of evolution.

De Frutos et al. recently published their surprising findings of longer telomeres in the sperms of younger mice compared to older mice [21]. What they found is actually the opposite of the human situation, namely that the offspring of younger males inherited longer leukocyte telomeres. Some of my critics have argued that this clearly shows that my evolutionary theory of transgenerational telomere erosion cannot be applied to the real world. Firstly, this was not a longitudinal study and the author’s conclusion of telomere erosion in the germ cells of male mice during aging is therefore not justified. Secondly, the study results have to be confirmed by others. Thirdly, I propose that a birth-cohort effect is the underlying cause, although in this special case the other way round. The authors performed their experiments on two mouse species, *Mus musculus* and *Mus spretus*. The aging effect on male germ telomeres was reported only for *M. musculus* (namely the lab strain C57/CBA). Most laboratory strains of *M. musculus* have a history of prolonged inbreeding and some have telomeres up to 100 kb long [22], 5–10 times the size of telomeres of humans and wild-derived mice (e.g. *Mus spretus*). Hemann and Greider already suggested in 2000 [22] that telomere elongation occurs as a function of extensive breeding in an isolated colony, although the mechanism for this elongation is unclear. Similarly, Manning and colleagues, based on their experimental findings in inbred strains of mice, concluded that prolonged inbreeding elongates telomeres [23]. These important experiments had been cited in my initial 2004 paper, where I postulated that a theory of transgenerational telomere erosion requires once in a while some kind of telomere reset and the underlying mechanism could be inbreeding [8]. Hence, I propose that the laboratory strain of *M. musculus* tested by de Frutos et al. is in a phase of transgenerational telomere elongation, which possibly takes place during the blastula stage of embryonic development through the ALT mechanism, activated by yet unknown effects of inbreeding [23]. In this special case, younger male mice, which were members of a more recent generation, underwent a higher number of generation-based telomere elongation events than their older male colleagues (=birth-cohort effect). Therefore, artificially bred animals like laboratory strains of *Mus musculus* might not be the best choice if someone wants to draw any conclusions related to standard telomere biology in mammals.

## Testing the hypothesis

Despite being the exclusive mainstream model, an increase in length of sperm telomeres during male aging has never been shown experimentally. To my knowledge, no one has ever measured the telomere length of sperm samples from the same individuals twice within, for example, 5 to 10 years. Thus, I suggest carrying out a longitudinal study on human sperm telomere length. Perhaps study participants of previous cross-sectional studies could be tested again, by the same investigators. I expect the telomere length of sperms to be stable in the same individuals over time (this also applies to the mouse strain tested by de Frutos et al.). Longitudinal studies, either on oocytes or sperms in humans or animals, are required to prove (or to falsify) the current mainstream model of the rejuvenation of the gonads of old men.

## Implications of the hypothesis

Transgenerational telomere erosion as a mechanism of species evolution definitely requires a paradigm shift [6]. A first step in the right direction would be to interpret the existing human data correctly and to accept the phenomenon of long-telomered spermatocytes in elderly men’s testes as what it obviously is: a birth-cohort effect and not any paradoxical rejuvenation of the gonads of old men. Because much of the current scientific literature – perhaps half – may simply be untrue [24], I urge for a reintroduction of theoretical reasoning, which, in the hands of capable investigators, might help to clean up the mess caused by current biological experimentation without proper theory [25].

## Abbreviations

ALT: alternative lengthening of telomeres
